# Exploration of Therapeutic Targets Using CDK4 Inhibitors for Head and Neck Mucosal Melanoma

**DOI:** 10.1002/ohn.70137

**Published:** 2026-01-22

**Authors:** Takayoshi Hattori, Makiko Fujii, Tsutomu Ueda, Akira Ishikawa, Yuichi Mine, Bingwen Xu, Tomoya Suehiro, Minoru Hattori, Hiroaki Tahara, Yuki Sato, Nobuyuki Chikuie, Takayuki Taruya, Takao Hamamoto, Takashi Ishino, Sachio Takeno

**Affiliations:** ^1^ Department of Otorhinolaryngology, Head and Neck Surgery, Graduate School of Biomedical and Health Sciences Hiroshima University Hiroshima Japan; ^2^ Department of Genomic Oncology, Oral Medicine, Graduate School of Biomedical and Health Sciences Hiroshima University Hiroshima Japan; ^3^ Department of Molecular Pathology, Graduate School of Biomedical and Health Sciences Hiroshima University Hiroshima Japan; ^4^ Department of Medical Systems Engineering, Graduate School of Biomedical and Health Sciences Hiroshima University Hiroshima Japan; ^5^ Department of Medical Education Institute of Biomedical & Health Sciences, Center for Medical Education Institute of Biomedical & Health Sciences Hiroshima University Hiroshima Japan

**Keywords:** cell line, cyclin‐dependent kinase 4, mucosal melanoma, growth inhibitors, head and neck, molecular‐targeted therapy

## Abstract

**Objective:**

Mucosal melanoma (MM) is an extremely aggressive malignant tumor in the head and neck region associated with a poor prognosis. In the present study, we conducted cell proliferation assay and western blotting using cell lines derived from MM and the immunohistochemical analysis of pathological MM tissues to identify novel therapeutic targets. Herein, we report on the potential of cyclin‐dependent kinase 4 (CDK4) inhibitors as molecular targeted therapies for MM.

**Study Design:**

Retrospective cohort study and laboratory analysis.

**Setting:**

A tertiary referral center.

**Methods:**

MTT assay and western blotting were performed on the HMV‐II (RCB0777) and GAK (JCRB0180) cell lines, treated with the CDK4 inhibitors abemaciclib (LY2835219) and palbociclib (PD‐0332991). This retrospective cohort study included patients with head and neck MM; immunohistochemistry was performed on clinical specimens.

**Results:**

Abemaciclib and palbociclib showed concentration‐dependent cytostatic effects on HMV‐II and GAK cells at 72 hours in the MTT assay. In western blotting, they exhibited concentration‐dependent inhibitory effects on phosphorylated RB1 in HMV‐II and GAK cells at 24 hours. Of 23 patients, 18 (78.3%) had positive results on CDK4 immunostaining. No clinicopathologic factors were significantly associated with CDK4 status.

**Conclusion:**

Abemaciclib and palbociclib may suppress MM cell proliferation. The CDK4 signaling pathway is a potential target for molecular‐targeted therapies in MM.

Mucosal melanoma (MM) is a highly aggressive malignant tumor of the head and neck region. It primarily develops from melanocytes located in the mucosa of the sinonasal sinuses, oral cavity, and pharynx.^1^, ^2^ The incidence of MM in the head and neck region accounts for 31% to 55% of all cases of MM.^3^, ^4^ Generally, MM has a significantly worse prognosis than cutaneous melanoma (CM), with a 5‐year survival rate of 14% for MM compared with 80% for CM.^5^, ^6^ Immunotherapy using immune checkpoint inhibitors (ICIs) has advanced for CM; nonetheless, patients with MM tend to be less responsive to ICIs.^7^ The carcinogenic factors associated with MM remain unclear. Ultraviolet radiation, a major risk factor for CM, is not directly associated with the development of MM.^8^

Recent studies, including whole genome and exome sequencing, next‐generation sequencing technologies, and bioinformatics analyses, have revealed specific molecular profiles and genetic alterations associated with MM.^1^, ^9^, ^10^ In particular, abnormal activation of the cyclin‐dependent kinase 4 (CDK4) signaling pathway has been observed.^1^, ^10^, ^11^ CDK4 interacts with D‐type cyclins to phosphorylate retinoblastoma (p‐RB) tumor suppressor proteins, promoting cell cycle progression from the G1 to S phase.^12^ These are crucial factors. Abnormal cell cycle progression and uncontrolled cell proliferation are hallmark characteristics of cancer.^13^, ^14^ CDK dysregulation is essential in tumorigenesis. Consequently, therapies targeting CDK4 have been clinically applied to various malignancies^15^, ^16^, ^17^, ^18^ and have gained significant interest.

Exploring the role of CDK4 and its signaling pathway in MM has become an important direction for developing new treatment strategies. In this study, we conducted a cell proliferation assay and western blot analysis using cell lines derived from MM cells to evaluate their response to CDK4 inhibitors. Immunohistochemical analysis of the pathological clinical tissues was conducted to assess CDK4 expression. Here, we report the potential of CDK4 inhibitors as targets for molecular‐targeted therapies.

## Methods

### Cell Culture and Reagents

HMV‐II (RCB0777) and GAK (JCRB0180) cell lines were purchased from Riken (Tsukuba, Japan). All MM‐derived cell lines were cultured in Ham's F‐12 with L‐Gln liquid medium (17458‐65; NACALAI TESQUE, Inc.) supplemented with 10% FBS at 37°C in a humidified atmosphere containing 5% CO₂. The cells were tested for mycoplasma contamination. Abemaciclib (LY2835219) and palbociclib (PD‐0332991) HCl were purchased from Selleck and were dissolved in ethanol and ultrapure water at 10 mM stock concentrations and used as inhibitors to block intracellular signaling. The cell proliferation assay was conducted using PrestoBlue™ HS Cell Viability Reagents (Thermo Fisher Scientific).

### Western Blotting and Antibodies

The cells were washed with ice‐cold PBS and lysed on ice for 10 min using a lysis buffer (10 mM HEPES, 200 mM NaCl, 30 mM sodium pyrophosphate, 50 mM NaF, 5 mM ZnCl_2_, and 1.0% TritonX‐100 [pH 7.5]). This buffer was supplemented with a Protease Inhibitor Cocktail (25955‐24; NACALAI TESQUE, Inc.). Cell lysates were centrifuged at 12,000*g* for 20 min, and the supernatants were collected for standard procedures. RB1 Polyclonal antibody (10048‐2‐Ig) and Phospho‐RB1 (Ser807/811) polyclonal antibody (30376‐1‐AP) as primary antibody were purchased from Proteintech Group, Inc. An anti‐actin antibody (MAB1501; Merck) was used as an enumeration control. Secondary antibody Anti‐rabbit IgG, HRP‐linked Antibody (#7074), and anti‐mouse (♯7076) were obtained from Cell Signaling Technology. Protein signals were detected using ImmunoStarR Zeta (295‐72404; FUJIFILM Wako). Band densities were quantified using the ImageJ (Fuji) densitometry analysis software (National Institutes of Health).

### Immunohistochemistry (IHC)

**Figure 1:**
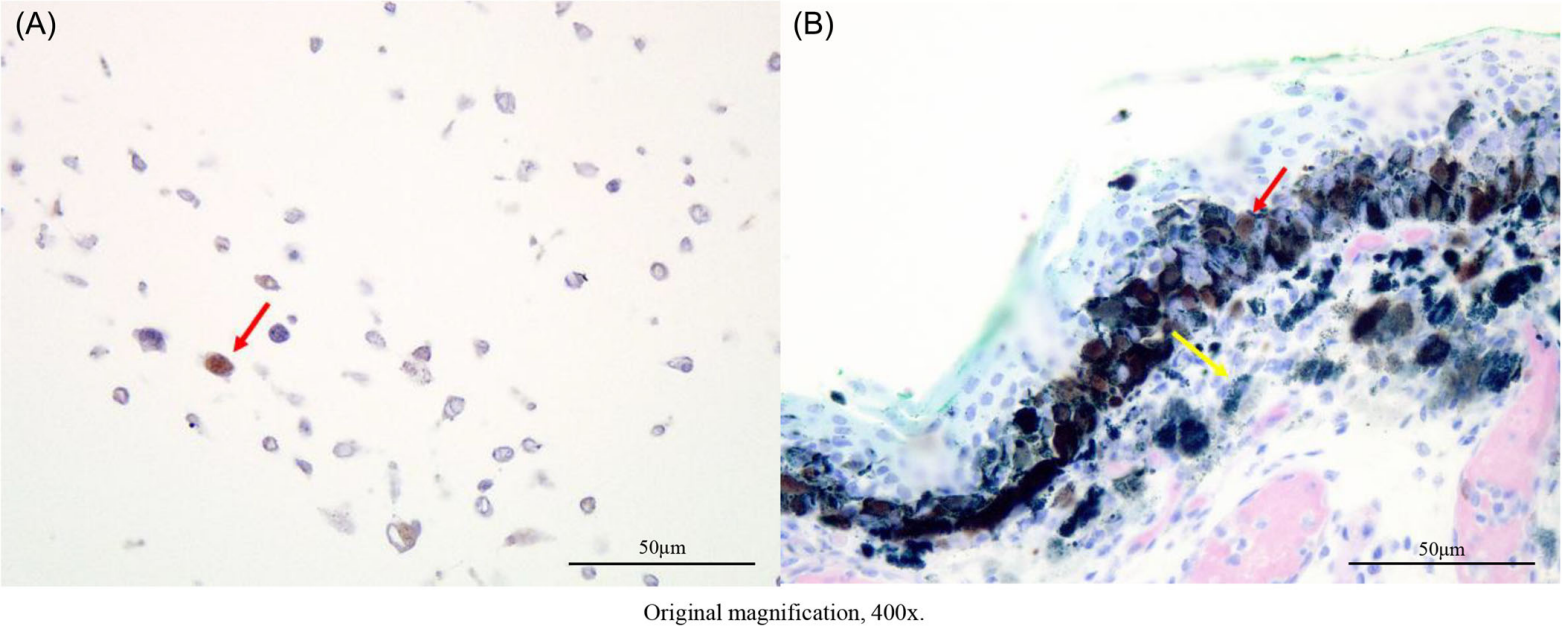
(A) CDK4 immunostaining for cell block. The CDK4 antibody stained the cell nuclei (red arrow). (B) CDK4 immunostaining for clinical specimens. Counterstaining was Giemsa, which stained melanin green (yellow arrow) and CDK4 brown (red arrow).

CDK4 Monoclonal antibody (CDK4 [D9G3E] rabbit mAb; #12790) was purchased from Cell Signaling Technology, Inc. First, we created cell blocks from the HMV‐II cell line, which was derived from MM, and confirmed that the antibody against CDK4 stained the cell nuclei (Figure 1A). HMV‐II was used as a positive control for CDK4 immunostaining in subsequent analyses of clinical specimens.

Next, IHC was conducted on formalin‐fixed paraffin‐embedded tissue sections from clinical specimens. Briefly, 4‐μm‐thick sections were cut and processed for IHC detection using the CDK4 Monoclonal antibody (CDK4 [D9G3E] Rabbit mAb #12790; Cell Signaling Technology). Antigen retrieval was conducted by autoclaving the sections in a pH 9 buffer. The primary antibody was diluted at a ratio of 1:400. For the color reaction, the sections were incubated with the Dako Liquid DAB+ Substrate Chromogen System (#K3468). The slides were then counterstained with Giemsa, which stains melanin green and CDK4 brown. This staining facilitates the assessment of true nuclear staining by distinguishing it from melanin pigmentation (Figure 1B).

We quantified CDK4 immunoreactivity using digital image analysis in Python with scikit‐image and OpenCV. Color separation used the Ruifrok‐Johnston rgb2hed deconvolution^19^ for color separation to generate nuclear and chromogen (DAB) channels. These channels were rescaled to 8‐bit grayscale and used for nuclear segmentation and CDK4 positivity scoring. The program displayed color overlays with bounding boxes for positive (red) and negative (green) nuclei. After visually confirming adequate segmentation, the program exported the total and CDK4‐positive nuclear counts, along with the overall positive rate.

Finally, a surgical pathologist (A.I.) reviewed the immunoreactivity of each specimen. Only clear nuclear reactions were considered positive, whereas ambiguous, perinuclear, and cytoplasmic staining were excluded. CDK4 expression was evaluated using the immunoreactive score (IRS) (Table 1). In our study, tumor slices that scored at least two points were classified as CDK4‐positive.

**Table 1:** Immunoreactive Score (IRS)

A (intensity)	B (Proportion of positive cells)	IRS (multiplication of A and B)
0: no color reaction	0: no positive cells	0‐1: negative
1: weak reaction	1: < 10% of positive cells	2‐3: weak positive
2: moderate reaction	2: 10‐50% positive cells	4‐8: moderate positive
3: strong reaction	3: 51‐80% positive cells	9‐12: strongly positive
	4: >80% positive cells	

### Patients

We conducted a retrospective cohort study involving patients with head and neck MM at Hiroshima University Hospital in Japan between April 1, 2006, and March 31, 2023. Blood tests were conducted before the primary therapy. The neutrophil/lymphocyte ratio, prognostic nutritional index, and Modified Glasgow Prognostic Score were used as indicators of nutritional status. Imaging studies, including body cavity computed tomography, magnetic resonance imaging, and positron emission tomography, were conducted as required.

All patients were followed up until their death or loss of contact. Data on baseline characteristics and clinical outcomes were collected from the clinical records. Overall survival (OS) and progression‐free survival (PFS) were analyzed. OS was calculated from the start of treatment until death or termination of the final follow‐up day. PFS was calculated from the start of treatment until disease progression, death, or discontinuation on the final follow‐up day. Additionally, we analyzed factors associated with CDK4 status.

The Institutional Review Board of Hiroshima University Hospital approved this study. It was conducted under the Declaration of Helsinki Ethical Principles for Medical Research Involving Human Subjects. The study protocol was posted at our institution. All patients were offered the opportunity to opt out of the study.

### Statistical Analysis

Continuous variables are reported as medians (ranges), and categorical variables are reported as proportions and/or percentages. Categorical data were analyzed using Fisher's exact test, and continuous data between groups were compared using the Mann‐Whitney *U* test. Univariate analyses of OS and PFS were conducted using Cox proportional hazards models. To investigate the association between patient characteristics and CDK4 status, we performed univariate logistic regression analysis. Statistical significance was defined as a *P* < .05. Calculations were conducted using SPSS version 27 (IBM).

## Results

### Cell Proliferation Assay (MTT Assay)

HMV‐II and GAK cells were seeded into 96‐well plates and treated with varying concentrations of abemaciclib and palbociclib as inhibitors. Each group included three technical replicates, and the experiment was independently repeated three times. After adding the inhibitors, the cells were cultured for 72 hours. PrestoBlue was added and incubated for 20 minutes, followed by measurements using a microplate reader. The results were subjected to statistical analyses.

**Figure 2:**
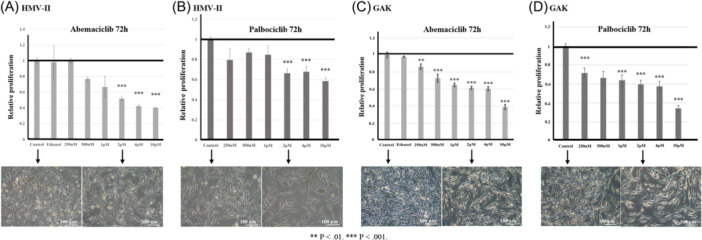
MTT assay for HMV‐Ⅱ and GAK. (A) HMV‐II treated with abemaciclib, (B) HMV‐II treated with palbociclib, (C) GAK treated with abemaciclib, (D) GAK treated with palbociclib. Pictures show a significant reduction in cell counts.

Abemaciclib and palbociclib showed concentration‐dependent cytostatic effects on HMV‐II cells at 72 h, with similar results observed in GAK cells (Figure 2).

### Western Blotting

HMV‐II and GAK cell lines were treated with abemaciclib and palbociclib as inhibitors at different concentrations (250 nM, 500 nM, 1 μM, and 2 μM) for 24 hours. After treatment, cells were lysed, and western blot analysis was conducted to detect protein expression using specific antibodies. The protein density of phosphorylated RB1 (p‐RB1) in the same samples was quantified, with RB1 serving as an internal control. Statistical analysis of protein density was conducted using a one‐way analysis of variance. Each experiment was performed independently and repeated three times.

**Figure 3:**
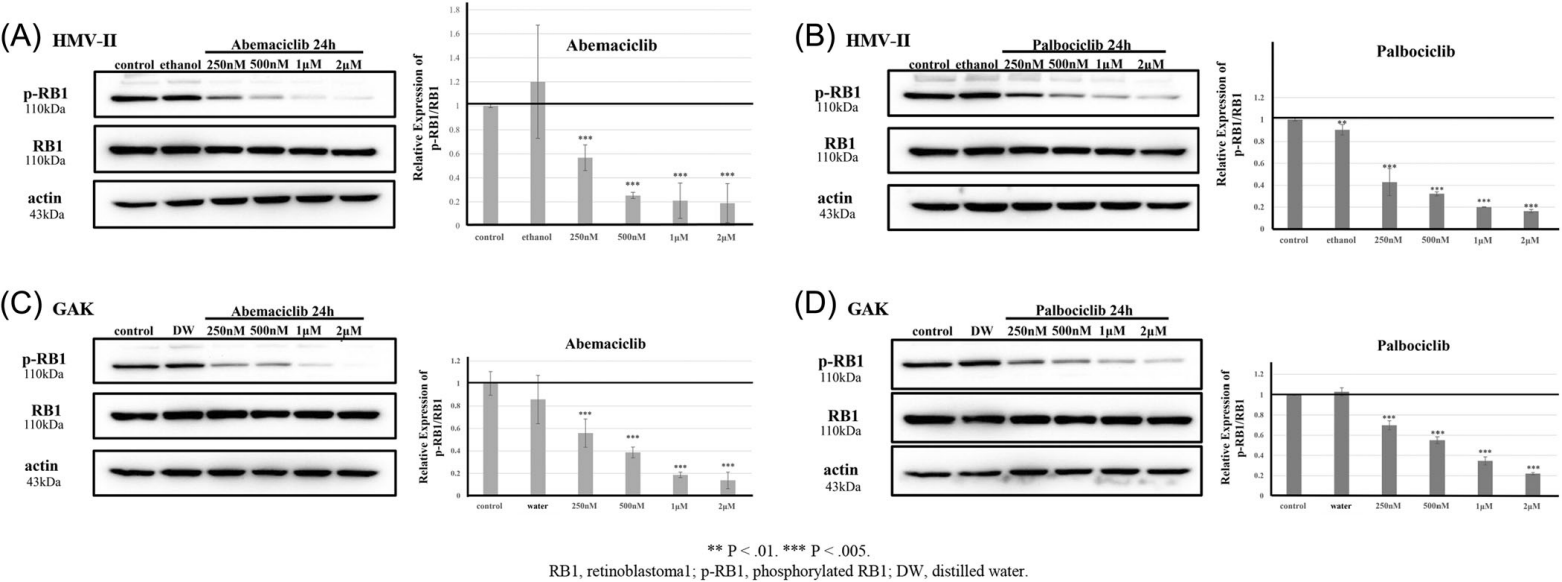
Western blotting for HMV‐II and GAK. (A) HMV‐II treated with abemaciclib, (B) HMV‐II treated with palbociclib, (C) GAK treated with abemaciclib, (D) GAK treated with palbociclib. Both inhibitors show concentration‐dependent inhibitory effects on p‐RB1.

Abemaciclib and palbociclib showed concentration‐dependent inhibitory effects on p‐RB1 in HMV‐II and GAK cells after 24 hours (Figure 3).

### Patient Characteristics

In this study, 23 patients were identified. Table 2 shows a summary of the patient characteristics. Twelve patients (52.2%) were male, and the median age was 74 (35‐98) years. The Eastern Cooperative Oncology Group Performance Status was 0 for 12 patients (52.2%) and 1 for 11 patients (47.8%). The most common primary site was the sinonasal sinus (69.6%, n = 16).

**Table 2:** Patient Characteristics

Characteristics	n = 23	%	Characteristics	n = 23	%	Characteristics	n = 23	%
Age, years			Recurrent or metastasis			Alanine aminotransferase (U/L)		
<70 years	8	34.8	No	7	30.4	<23	16	69.6
≥70 years	15	65.2	Recurrent	6	26.1	≥23	7	30.4
Sex			Metastasis	10	43.5	Lactate dehydrogenase (U/L)		
Male	12	52.2	Secondary therapy			<222	17	73.9
Female	11	47.8	Operation	4	17.4	≥222	6	26.1
BMI (body mass index)			Chemotherapy	9	39.1	Total protein (g/dL)		
≥18.5, <25	16	69.6	Heavy ion radiotherapy	1	4.3	<6.6	4	17.4
≥25	7	30.4	Radiation therapy	0	0	≥6.6	19	82.6
ECOG PS			Interferon	1	4.3	Albumin (g/dL)		
0	12	52.2	None	8	34.8	<4.1	8	34.8
1	11	47.8	Adjuvant nivolumab			≥4.1	15	65.2
Primary site			No	18	78.3	Blood urea nitrogen (mg/dL)		
Sinonasal sinus	16	69.6	Yes	5	21.7	<20	21	91.3
Oral	2	8.7	White blood cells (/μL)			≥20	2	8.7
Pharynx	1	4.3	<8600	22	95.7	Creatinine (mg/dL)		
Others	4	17.4	≥8600	1	4.3	<1.07	22	95.7
Clinical T Stage (UICC 8th)			Neutrophils (/μL)			≥1.07	1	4.3
T3	12	52.2	<3000	7	30.4	e‐GFR (mL/min)		
T4a	9	39.1	≥3000	16	69.6	<60	4	17.4
T4b	2	8.7	Lymphocytes (/μL)			≥60	19	82.6
Clinical Stage (UICC 8th)			<1000	4	17.4	C‐reactive protein (mg/dL)		
Ⅲ	11	47.8	≥1000	19	82.6	<0.14	11	47.8
Ⅳ	12	52.2	Eosinophils (/μL)			≥0.14	12	52.2
CDK4 status			<100	10	43.5	Neutrophil/lymphocyte ratio		
Negative	5	21.7	≥100	13	56.5	<2.2	10	43.5
Positive	18	78.3	Hemoglobin (g/dL)			≥2.2	13	56.5
CDK4 Immunoreactive score			<13.7	14	60.9	Prognostic nutritional index		
Negative	5	21.7	≥13.7	9	39.1	<45	2	8.7
Weak	10	43.5	Platelet count (×103/μL)			≥45	21	91.3
Moderate	5	21.7	<158	3	13.1	Modified GPS		
Strong	3	13.1	≥158	20	86.9	0	18	78.3
Primary therapy			Total bilirubin (mg/dL)			1	3	13.1
Operation	12	52.2	<1.5	23	100	2	2	8.7
Chemotherapy	0	0	≥1.5	0	0			
Heavy ion radiotherapy	6	26.1	Aspartate aminotransferase (U/L)					
Radiation therapy	3	13.1	<30	19	82.6			
Interferon	1	4.3	≥30	4	17.4			
None	1	4.3						

Abbreviations: ECOG PS, ECOG performance status; e‐GFR, estimated glomerular filtration rate; modified GPS, Modified Glasgow Prognostic Score.

CDK4 immunostaining was positive in 18 patients (78.3%), of whom 10 (43.5%) showed weak staining, five (21.7%) showed moderate staining, and three (13.1%) showed strong staining. The primary therapies were as follows: surgery in 12 (52.2%), heavy‐ion radiotherapy in 6 (26.1%), radiation therapy in 3 (13.1%), and interferon in 1 (4.3%), with no patient undergoing chemotherapy. Six patients (26.1%) had local recurrence, and 10 (43.5%) had distant metastases. One patient who did not receive the primary therapy was excluded from the efficacy analysis.

### Association of CDK4 Status with Characteristics

Factors associated with the CDK4 status were also evaluated. A univariate analysis was conducted to determine the background characteristics of each patient. No significant differences were observed in any patient characteristics (Supplemental Table S1, available online). Multivariate analysis was not conducted because the univariate analysis showed no significant differences.

Supplemental Tables S2 and S3, available online, show details of the analysis of prognostic factors for survival. The only factor that showed a statistically significant difference in OS was the clinical T stage. Only the presence or absence of recurrence or metastasis showed a statistically significant difference in PFS. CDK4 status did not differ significantly between OS and PFS.

## Discussion

Nassar et al^10^ compared MM meta‐analyses from 65 studies and CM analyses from The Cancer Genome Atlas. BRAF mutations account for 52% of CMs; however, they appear in only 6% of MM cases. This low frequency of BRAF mutations, a common therapeutic target in CM, is likely the reason for the low response rates to BRAF and MEK inhibitors in MM, and long‐term disease control is rarely achieved.^20^ For identifying effective treatment targets, a better understanding of the molecular biology of MM is essential.

Conversely, mutations in the p16‐cyclin D‐CDK4/6‐Rb protein pathway have been reported in MM.^21^, ^22^, ^23^ Abnormalities in CDK4 and aberrant activation of the CDK4 signaling pathway have been confirmed.^1^, ^10^, ^11^ Zhou et al.^9^ found that amplification of the 12q13‐15 locus, including the CDK4 region, is the most commonly altered genomic region in MM.

In the cell cycle, a single mother cell divides to produce two daughter cells. In normal cells, the cell cycle is negatively regulated by the checkpoint mechanism during the transition from G1 to S phase, G2 to M phase, and metaphase to anaphase. These checkpoints help control unregulated cell division. Notably, the checkpoint between the G1 and S phases, known as the restriction point, is the rate‐limiting step of the cell cycle.^24^ The progression through the restriction point involves forming and activating a complex between CDK4 and cyclin D1, which drives cell cycle progression. In cancer cells, excessive activation of division‐promoting signals, including membrane‐type growth factor signals or abnormalities at the restriction point, causes CDK4 to form complexes with cyclin D1. This leads to phosphorylation of the RB protein, which initiates the cell cycle and leads to uncontrolled proliferation.^25^

CDK4 inhibitors suppress cancer cell proliferation by inhibiting CDK4 and controlling its restriction point, thereby halting cell cycle progression. In clinical practice, CDK4‐targeted therapies have been applied to breast cancer and have recently gained significant interest from patients, physicians, and scientists.^15^, ^16^, ^17^, ^18^

In this study, CDK4 expression was confirmed through immunostaining of head and neck MM. However, its expression did not significantly correlate with OS or PFS. In breast cancer, CDK4 inhibitors have been proven to be effective and have been implemented in clinical practice based on international randomized phase III trials that showed prolonged PFS as the primary endpoint.^26^, ^27^, ^28^, ^29^ Nevertheless, some studies have suggested that CDK4 expression is not associated with efficacy, such as OS or PFS in breast cancer,^30^, ^31^ and no prognostic biomarkers have been identified. CDK4 expression itself is not associated with OS or PFS; nevertheless, CDK4 inhibitors have been shown to prolong OS and PFS in real‐world clinical practice.^32^, ^33^ Similarly, in MM, CDK4 expression itself is not associated with OS or PFS; however, CDK4 inhibitors may exhibit similar beneficial effects.

In this study, cell proliferation assays using HMV‐II (RCB0777) and GAK (JCRB0180) showed that abemaciclib and palbociclib inhibited cell proliferation. However, these cell lines were derived from vaginal and vulvar MM. Vaginal and vulvar MM has an incidence of <0.5 cases per million women annually, accounting for <1% of all melanomas in females (representing 15%‐20% of MMs globally).^34^ To our knowledge, no studies have specifically examined the relationship between CDK4 expression and OS or PFS in vaginal and vulvar MM. No unified consensus exists regarding the factors associated with OS or PFS.^35^, ^36^, ^37^ Given the reported molecular similarity between vaginal and nasal MMs,^38^ we used only available vaginal and vulvar mucosal‐derived cell lines. In this experiment, the administration of CDK4 inhibitors suppressed CDK4 and inhibited cell proliferation in these lines. In other words, CDK4 can be inhibited in breast cancer cells and MM cells. Given the effectiveness of CDK4 inhibitors in breast cancer, even in cases in which CDK4 expression is not associated with OS or PFS, these inhibitors may also be effective in treating head and neck MM. Given the clinical potential of targeting CDK4 in MM, the next important direction for therapeutic development is to explore the role of CDK4 and its signaling pathways in MM.

We did not use cell lines derived from CM; however, head and neck mucosal‐derived cell lines would be ideal for more precise experiments. These cell lines exist^39^; nonetheless, they are not currently in practical use and were difficult to obtain for this study. We are currently developing new head and neck MM cell lines.

A previous study has results different from those in this study, reporting that CDK4 expression was associated with 3‐year survival rates in 54 cases of MM (44 in the head and neck, six in the gastrointestinal tract, three in the genitourinary tract, and one in the lung).^40^ As mentioned earlier, no prognostic biomarkers have been identified for vaginal and vulvar MM or for breast cancer where CDK4 inhibitors are in practical use. We believe that further accumulation of cases and studies is necessary to identify biomarkers for MM, including those in the head and neck region.

It should be noted that the limitations of this study include the small sample size and the heterogeneity of primary treatment.

Next, we plan to conduct RNA sequencing to confirm the details of cellular regulation and investigate potential targets for further molecular‐targeted therapies.

## Conclusion

We conducted a cell proliferation assay and western blotting using cell lines derived from MM and confirmed CDK4 expression in MM pathological tissues through immunohistochemical analysis. Abemaciclib and palbociclib suppress RB phosphorylation and inhibit cell proliferation. The CDK4 signaling pathway is a promising target for molecular therapies in MM. Further validation in head and neck‐derived models is warranted.

## Author Contributions

**Takayoshi Hattori:** conceptualization, methodology analysis, interpreted the data, investigation, writing draft manuscript, review and editing; **Makiko Fujii:** conceptualization, methodology analysis, interpreted the data, investigation, review and editing; **Tsutomu Ueda:** conceptualization, methodology analysis, interpreted the data, investigation, review and editing; **Akira Ishikawa:** methodology analysis, interpreted the data, investigation, review and editing; **Yuichi Mine:** methodology analysis, interpreted the data, investigation, review and editing; **Bingwen Xu:** methodology analysis, interpreted the data, investigation, review and editing; **Tomoya Suehiro:** methodology analysis, interpreted the data, investigation, review and editing; **Minoru Hattori:** methodology analysis, interpreted the data, review and editing; **Hiroaki Tahara:** methodology analysis, investigation, review and editing; **Yuki Sato:** methodology analysis, investigation, review and editing; **Nobuyuki Chikuie:** methodology analysis, investigation, review and editing; **Takayuki Taruya:** methodology analysis, investigation, review and editing; **Takao Hamamoto:** methodology analysis, investigation, review and editing; **Takashi Ishino:** methodology analysis, investigation, review and editing; **Sachio Takeno:** methodology analysis, interpreted the data, review and editing, supervision.

## Disclosures

### Competing interests

None.

### Funding source

None.

## Supporting information

Supplemental Table S1. Univariate analyses for factors associated with CDK4 status. No significant differences were observed in any patient characteristics.
Supplemental Table S2. Univariate analyses for factors associated with overall survival. The only factor that showed a statistically significant difference was the clinical T stage. CDK4 status did not differ significantly.
Supplemental Table S3. Univariate analyses for factors associated with progression free survival. Only the presence or absence of recurrence or metastasis showed a statistically significant difference. CDK4 status did not differ significantly.
