# Supplementary material for: Exploration of Therapeutic Targets Using CDK4 Inhibitors for Head and Neck Mucosal Melanoma
Source: Otolaryngol Head Neck Surg. 2026 Jan 22;174(3):784–91. doi: 10.1002/ohn.70137 (PMC12948400; doi:10.1002/ohn.70137)
Supplement: Supplementary file 1 — Supplemental Table S1. Univariate analyses for factors associated with CDK4 status. No significant differences were observed in any patient characteristics. Supplemental Table S2. Univariate analyses for factors associated with overall survival. The only factor that showed a statistically significant difference was the clinical T stage. CDK4 status did not differ significantly. Supplemental Table S3. Univariate analyses for factors associated with progression free survival. Only the presence or absence of recurrence or metastasis showed a statistically significant difference. CDK4 status did not differ significantly. [file OHN-174-784-s001.docx]

| **Supplemental Table S1**. Univariate analyses for factors associated with CDK4 status | | | | | | | | | | |
| --- | --- | --- | --- | --- | --- | --- | --- | --- | --- | --- |
| Characteristics |  | CDK4 negative | CDK4 positive | P value | Characteristics |  | CDK4 negative | CDK4 positive | P value |  |
|  |  | n (%) | n (%) |  |  |  | n (%) | n (%) |  |  |
| ECOG PS | 0 | 3 (60.0) | 8 (47.1) | 1 | Aspartate aminotransferase (U/L) | <30 | 5 (100.0) | 13 (76.5) | .535 |  |
|  | 1 | 2 (40.0) | 9 (52.9) |  |  | ≥30 | 0 (0.0) | 4 (25.6) |  |  |
| Primary site | Sinonasal sinus | 4 (80.0) | 12 (70.6) | 1 | Alanine aminotransferase (U/L) | <23 | 5 (100.0) | 10 (58.8) | .135 |  |
|  | Oral | 0 (0.0) | 2 (11.8) |  |  | ≥23 | 0 (0.0) | 7 (41.2) |  |  |
|  | Pharynx | 0 (0.0) | 1 (5.9) |  | Lactate dehydrogenase (U/L) | <222 | 4 (80.0) | 13 (76.5) | 1 |  |
|  | Others | 1 (20.0) | 2 (11.8) |  |  | ≥222 | 1 (20.0) | 4 (23.5) |  |  |
| Clinical T Stage | T3 | 3 (60.0) | 9 (52.9) | 1 | Total protein (g/dL) | <6.6 | 1 (20.0) | 3 (17.6) | 1 |  |
| (UICC 8th) | T4a | 2 (40.0) | 7 (41.2) |  |  | ≥6.6 | 4 (80.0) | 14 (82.4) |  |  |
|  | T4b | 0 (0.0) | 1 (5.9) |  | Albumin(g/dL) | <4.1 | 2 (40.0) | 5 (29.4) | 1 |  |
| Clinical Stage | Ⅲ | 3 (60.0) | 8 (47.1) | 1 |  | ≥4.1 | 3 (60.0) | 12 (70.6) |  |  |
| (UICC 8th) | Ⅳ | 2 (40.0) | 9 (52.9) |  | Blood urea nitrogen (mg/dL) | <20 | 5 (100.0) | 15 (88.2) | 1 |  |
| Primary therapy | Operation | 3 (60.0) | 9 (52.9) | 1 |  | ≥20 | 0 (0.0) | 2 (11.8) |  |  |
|  | Heavy ion radiotherapy | 1 (20.0) | 5 (29.4) |  | Creatinine (mg/dL) | <1.07 | 5 (100.0) | 16 (94.1) | 1 |  |
|  | Radiation therapy | 1 (20.0) | 2 (11.8) |  |  | ≥1.07 | 0 (0.0) | 1 (5.9) |  |  |
|  | Interferon | 0 (0.0) | 1 (5.9) |  | e-GFR (mL/min) | <60 | 1 (20.0) | 3 (17.6) | 1 |  |
| Recurrent or Metastasis | No | 2(40.0) | 4 (23.5) | .585 |  | ≥60 | 4 (80.0) | 14 (82.5) |  |  |
|  | Yes | 3(60.0) | 13 (76.5) |  | C-reactive protein(mg/dL) | <0.14 | 1 (20.0) | 10 (58.8) | .311 |  |
| Adjuvant nivolumab | No | 3 (60.0) | 14 (82.4) | .548 |  | ≥0.14 | 4 (80.0) | 7 (41.2) |  |  |
|  | Yes | 2 (40.0) | 3 (17.6) |  | Neutrophil/lymphocyte ratio | <2.2 | 2 (40.0) | 7 (41.2) | 1 |  |
| White Blood Cells (/μL) | <8600 | 5 (100.0) | 16 (94.1) | 1 |  | ≥2.2 | 3 (60.0) | 10 (58.8) |  |  |
|  | ≥8600 | 0 (0.0) | 1 (5.9) |  | Modified GPS | 0 | 3 (60.0) | 15 (88.2) | .210 |  |
| Hemoglobin (g/dL) | <13.7 | 4 (80.0) | 9 (52.9) | .360 |  | 1 | 1 (20.0) | 2 (11.8) |  |  |
|  | ≥13.7 | 1 (20.0) | 8 (47.1) |  |  | 2 | 1 (20.0) | 0 (0.0) |  |  |
| Platelet count(×103/μL) | <158 | 0 (0.0) | 3 (17.6) | 1 |  |  |  |  |  |  |
|  | ≥158 | 5 (100.0) | 14 (82.4) |  |  |  |  |  |  |  |
| Abbreviation: ECOG PS, ECOG performance status; e-GFR, estimated glomerular filtration rate; Modified GPS, Modified Glasgow Prognostic Score. | | | | | | | | | | |

| **Supplemental Table S2**. Univariate analyses for factors associated with overall survival | | | | | |
| --- | --- | --- | --- | --- | --- |
| Parameters | Category | n (%) | HR (95% CI) | P value |  |
| ECOG PS | 0 | 11 (50.0) | 2834 (539-NA) | .254 |  |
|  | 1 | 11 (50.0) | 966 (259-NA) |  |  |
| Primary site | Sinonasal sinus | 16 (72.7) | 1278 (274-2971) | .719 |  |
|  | Oral | 2 (9.1) | 347 (347-NA) |  |  |
|  | Pharynx | 1 (4.5) | NA (NA-NA) |  |  |
|  | Others | 3 (13.6) | NA (759-NA) |  |  |
| Clinical T Stage | T3 | 12 (54.5) | 2834 (539-NA) | .012 |  |
| (UICC 8th) | T4a | 9 (40.9) | 1278 (217-NA) |  |  |
|  | T4b | 1 (4.5) | 259 (NA-NA) |  |  |
| Clinical Stage | Ⅲ | 11 (50.0) | 2971 (539-NA) | .249 |  |
| (UICC 8th) | Ⅳ | 11 (50.0) | 1278 (259-NA) |  |  |
| Primary therapy | Operation | 12 (54.5) | 2971 (347-NA) | .585 |  |
|  | Heavy ion radiotherapy | 6 (27.3) | 1278 (259-NA) |  |  |
|  | Radiation therapy | 3 (13.6) | NA (274-NA) |  |  |
|  | Interferon | 1 (4.5) | NA (NA-NA) |  |  |
| Recurrent or Metastasis | No | 6 (27.3) | NA (660-NA) | .271 |  |
|  | Yes | 16 (72.7) | 1278 (347-NA) |  |  |
| Adjuvant nivolumab | No | 17 (77.3) | 2834 (347-NA) | .961 |  |
|  | Yes | 5 (22.7) | 759 (660-NA) |  |  |
| CDK4 status | Negative | 5 (22.7) | 966 (660-NA) | .560 |  |
|  | Positive | 17 (77.3) | 2834 (347-NA) |  |  |
| CDK4 Immunoreactive score 1 | Negative | 5 (33.7) | 966 (660-NA) | .737 |  |
|  | Weak | 9 (40.9) | NA (274-NA) |  |  |
|  | Moderate | 5 (22.7) | 2124.5 (259-NA) |  |  |
|  | Strong | 3 (13.6) | 2834 (217-NA) |  |  |
| CDK4 Immunoreactive score 2 | Negative+Weak | 14 (63.6) | NA (539-NA) | .336 |  |
|  | Moderate+Strong | 8 (36.4) | 2834 (539-NA) |  |  |
| Abbreviation: ECOG PS, ECOG performance status; HR, hazard ratio; CI, confidence interval. | | | | | |

| **Supplemental Table S3**. Univariate analyses for factors associated with progression free survival | | | | | |
| --- | --- | --- | --- | --- | --- |
| Parameters | Category | n (%) | HR (95% CI) | P value |  |
| ECOG PS | 0 | 11 (50.0) | 456.5 (128-NA) | .906 |  |
|  | 1 | 11 (50.0) | 321 (69-2568) |  |  |
| Primary site | Sinonasal sinus | 16 (72.7) | 702 (162-2749) | .282 |  |
|  | Oral | 2 (9.1) | 202 (NA-NA) |  |  |
|  | Pharynx | 1 (4.5) | 291 (NA-NA) |  |  |
|  | Others | 3 (13.6) | 321 (131-NA) |  |  |
| Clinical T Stage | T3 | 12 (54.5) | 338 (202-2568) | .324 |  |
| (UICC 8th) | T4a | 9 (40.9) | 575 (48-NA) |  |  |
|  | T4b | 1 (4.5) | 162 (NA-NA) |  |  |
| Clinical Stage | Ⅲ | 11 (50.0) | 520 (202-2568) | .692 |  |
| (UICC 8th) | Ⅳ | 11 (50.0) | 202 (69-NA) |  |  |
| Primary therapy | Operation | 12 (54.5) | 321 (131-NA) | .456 |  |
|  | Heavy ion radiotherapy | 6 (27.3) | 575 (128-NA) |  |  |
|  | Radiation therapy | 3 (13.6) | NA (69-NA) |  |  |
|  | Interferon | 1 (4.5) | 202 (NA-NA) |  |  |
| Recurrent or Metastasis | No | 6 (27.3) | NA (NA-NA) | .003 |  |
|  | Yes | 16 (72.7) | 247 (131-575) |  |  |
| Adjuvant nivolumab | No | 17 (77.3) | 338 (131-734) | .253 |  |
|  | Yes | 5 (22.7) | NA (291-NA) |  |  |
| CDK4 status | Negative | 5 (22.7) | 702 (338-NA) | .398 |  |
|  | Positive | 17 (77.3) | 291 (131-2568) |  |  |
| CDK4 Immunoreactive score 1 | Negative | 5 (33.7) | 702 (338-NA) | .360 |  |
|  | Weak | 9 (40.9) | 202 (69-321) |  |  |
|  | Moderate | 5 (22.7) | 1571.5 (162-NA) |  |  |
|  | Strong | 3 (13.6) | 2749 (48-NA) |  |  |
| CDK4 Immunoreactive score 2 | Negative+Weak | 14 (63.6) | 306 (131-734) | .375 |  |
|  | Moderate+Strong | 8 (36.4) | 2568 (48-NA) |  |  |
| Abbreviation: ECOG PS, ECOG performance status; HR, hazard ratio; CI, confidence interval. | | | | | |
